# Preoperative Sonographic Prediction of Limited Axillary Disease in Patients with Primary Breast Cancer Meeting the Z0011 Criteria: an Alternative to Sentinel Node Biopsy?

**DOI:** 10.1245/s10434-022-11829-1

**Published:** 2022-04-29

**Authors:** Julia Caroline Radosa, Erich-Franz Solomayer, Martin Deeken, Peter Minko, Julia Sarah Maria Zimmermann, Askin Canguel Kaya, Marc Philipp Radosa, Lisa Stotz, Sarah Huwer, Carolin Müller, Maria Margarete Karsten, Gudrun Wagenpfeil, Christoph Georg Radosa

**Affiliations:** 1 Department of Gynaecology and Obstetrics, Saarland University Hospital, Homburg, Saar, Germany; 2 Department of Gynaecology and Obstetrics, Knappschaftsklinikum Puettlingen, Puettlingen, Germany; 3 Department for Diagnostic and Interventionel Radiology, Duesseldorf University Hospital, Duesseldorf, Germany; 4 Department of Gynaecology & Obstetrics, Klinikum Bremen-Nord, Bremen, Germany; 5 Charité – University Medicine Berlin, Corporate Member of Freie University Berlin, Humboldt-Universität zu Berlin, Berlin, Germany; 6 Department of Gynecology with Breast Center, Berlin Institute of Health, Berlin, Germany; 7 Institute of Medical Biometry, Epidemiology and Medical Informatics, Saarland University Hospital, Homburg, Saar, Germany; 8 Department of Diagnostic and Interventional Radiology, University Hospital Carl Gustav Carus, TU Dresden, Dresden, Germany

## Abstract

**Purpose:**

To assess the accuracy of preoperative sonographic staging for prediction of limited axillary disease (LAD, one or two metastatic lymph nodes) and to identify factors associated with high prediction–pathology concordance in patients with early-stage breast cancer meeting the Z0011 criteria.

**Materials and Methods:**

Patients treated between January 2015 and January 2020 were included in this retrospective, multicentric analysis of prospectively acquired service databases. The accuracy of LAD prediction was assessed separately for patients with one and two suspicious lymph nodes on preoperative sonography. Test validity outcomes for LAD prediction were calculated for both groups, and a multivariate model was used to identify factors associated with high accuracy of LAD prediction.

**Results:**

Of 2059 enrolled patients, 1513 underwent sentinel node biopsy, 436 primary and 110 secondary axillary dissection. For LAD prediction in patients with one suspicious lymph node on preoperative ultrasound, sensitivity was 92% (95% CI 87–95%), negative predictive value (NPV) was 92% (95% CI 87–95%), and the false-negative rate (FNR) was 8% (95% CI 5–13%). For patients with two preoperatively suspicious nodes, the sensitivity, NPV, and FNR were 89% (95% CI 84–93%), 73% (62–83%), and 11% (95% CI 7–16%), respectively. On multivariate analysis, the number of suspicious lymph nodes was associated inversely with correct LAD prediction ([OR 0.01 (95% CI 0.01–0.93), *p* ≤ 0.01].

**Conclusions:**

Sonographic axillary staging in patients with one metastatic lymph node predicted by preoperative ultrasound showed high accuracy and a false-negative rate comparable to sentinel node biopsy for prediction of limited axillary disease.

The approach to axillary surgery for patients with breast cancer has evolved tremendously over recent decades, leading to significant changes in the role of preoperative axillary imaging.^1,2^ Sentinel node biopsy (SNB) has replaced axillary lymph node dissection (ALND) for primary surgical staging of clinical node-negative breast cancer patients, a paradigm shift that made the identification of axillary involvement the main goal of preoperative axillary staging as these patients could bypass SNB and proceed directly to ALND.^3^ In addition, large prospective trials, such as the American College of Surgeons Oncology Group Z0011 trial, have shown that ALND can be safely omitted in patients with early-stage (T1–2) breast cancer who undergo breast-conserving therapy and in whom SNB reveals two or fewer metastatic lymph nodes.^4–6^ With the implementation of these findings into clinical practice, the use of axillary imaging became controversial from a surgical standpoint, as preoperative detection of metastatic axillary disease would process patients directly to ALND, although these patients might not have been candidates for ALND following SNB according to the Z0011 protocol. As the mere preoperative identification of axillary disease was no longer sufficient to triage patients with early-stage breast cancer to appropriate axillary surgical treatment, a clinical need for preoperative quantification of the extent of axillary disease arose. Consecutively, preoperative breast imaging studies focused on the distinction of patients with limited axillary disease (LAD, one or two metastatic nodes) who would not undergo ALND after a positive sentinel due to the lack of therapeutic implication from patients with extensive axillary disease (EAD, three or more metastatic lymph nodes) who would benefit from ALND. These studies revealed a correlation between the number of abnormal lymph nodes identified by preoperative axillary ultrasound and the number of metastatic nodes on final pathology.^7–9^ Given these findings, the recent NCCN Clinical Practice Guidelines in Oncology (NCCN guidelines) advise consideration of SNB for patients meeting the Z0011 criteria in whom two or fewer suspicious lymph nodes are found on preoperative imaging, even when one node shows biopsy-proven positivity.^10^ If limited histopathological axillary disease could be identified safely by preoperative ultrasound and core needle biopsy, SNB may constitute overtreatment and omission of SNB in this setting due to a lack of consequence in selected patient populations might be an approach for further scientific evaluation. The purposes of this study were to assess the accuracy of preoperative sonographic axillary staging for prediction of LAD (one or two metastatic nodes on final pathology) in patients with early-stage breast cancer meeting the Z0011 criteria, who underwent ALND, and to identify factors associated with high concordance between sonographic prediction and histopathology.

## Materials and Methods

### Patient Selection

The study was approved by the institutional review committee and met the guidelines of their responsible governmental agency. The requirement for participants’ written informed consent was waived. All patients treated for primary breast cancer between January 2015 and January 2020 at one of the two study sites were identified retrospectively from prospectively maintained breast service databases. All patients who underwent preoperative ultrasound including axillary staging and surgery at one of the participating sites were eligible for inclusion in this study. Patients who completed neoadjuvant chemotherapy before axillary surgery were excluded. Other exclusion criteria were pathological T3/T4 or metastatic disease and incomplete clinical data. Breast cancer subtypes were defined by immunohistochemistry (IHC) as luminal A (positive expression of estrogen and/or progesterone receptor, lack of HER2/neu overexpression, and ki67 < 15%), luminal B (positive expression of estrogen and/or progesterone receptor, lack of HER2/neu overexpression, and ki67 ≥ 15%), HER2/neu positive (positive/negative expression of estrogen or progesterone receptor and HER2/neu overexpression (defined as 3+ expression or 2+ expression by IHC and a ratio of ≥ 2.0 by fluorescence in situ hybridization)), and triple negative (absence of estrogen and progesterone receptor expression and lack of HER-2/neu overexpression (defined as 1+ expression or 2+ expression by IHC and a ratio of < 2.0 by fluorescence in situ hybridization)). Data used in this study, including treatment and outcome data, were extracted from the patients’ medical records.

### Preoperative Ultrasound

Four breast imaging professionals with 5–25 years of experience in breast imaging performed the preoperative ultrasound examinations independently using Voluson E8/10 (GE Healthcare, Chicago, IL, USA) and Hitachi Hi Vision Ascendus (Hitachi, Tokyo, Japan) devices equipped with 5–12 MHz and 13–3 MHz linear-array transducers. These examinations were performed a median of 13 (range 8–27) days before surgery. According to institutional standard, sonographic evaluation of the axilla was performed routinely in all breast cancer patients as part of the diagnostic workup and included standardized assessment of level I, II, III, internal mammary, supra-, and infraclavicular lymph nodes.^11–13^ In cases of sonographic suspicion of axillary nodal involvement (defined as rounded hypoechoic lymph node, focal cortical bulging or eccentric cortical thickening, complete or partial effacement of the fatty hilum, or replacement of a lymph node with an irregular mass), axillary biopsy (core needle biopsy) of the most suspicious node was performed.^12–15^ The number of axillary nodes suspected to be metastatic on ultrasound examination was recorded. Patients with suspicious lymph nodes other than axillary lymph nodes were excluded from the analysis.

Preoperative axillary staging was defined as positive when preoperative ultrasound showed suspicion of axillary nodal involvement and this suspicion was confirmed by percutaneous biopsy. Negative preoperative axillary staging was defined as either absence of nodal involvement on preoperative ultrasound or suspicion of axillary nodal involvement on preoperative ultrasound but negative axillary core needle biopsy.

Patients with negative preoperative axillary staging proceeded to sentinel node biopsy. Patients with positive preoperative axillary staging proceeded to ALND. Secondary ALND was also performed in all patients with positive SNB findings. LAD was defined as presence of one or two metastatic axillary lymph nodes. EAD was defined as presence of three or more metastatic axillary lymph nodes. Pathological staging was performed according to the 8th edition of the American Joint Committee on Cancer’s cancer staging manual.^15^

### Data Analysis

Preoperative sonographic axillary findings were compared with final pathology for prediction of absence of nodal involvement (N0) in patients undergoing SNB and for number of metastatic lymph nodes in patients undergoing ALND. Patients with preoperative suspicion of LAD were assessed separately. The accuracy of preoperative sonographic axillary staging for identification of patients with histopathological LAD (≤ 2 metastatic axillary lymph nodes on final pathology) was assessed separately in patients with ultrasound-detected LAD undergoing ALND.

This group was further divided according to number of predicted metastatic lymph nodes (one or two) on preoperative ultrasound. The first group (group 1) consisted of patients with one suspected metastatic lymph nodes on preoperative ultrasound and ≤ 2 metastatic lymph nodes on final pathology, and the second group (group 2) of patients with sonographic suspicion of two metastatic lymph node and ≤ 2 metastatic lymph nodes in final pathology.

### Statistical Analysis

The accuracy, sensitivity, specificity, positive predictive value (PPV), negative predictive value (NPV), false-negative rate (FNR), and false-positive rate (FPR) were calculated for the sonographic prediction of N0 disease in patients undergoing SNB and for the two groups with LAD (group 1: one sonographically predicted metastatic lymph nodes/≤ 2 metastatic lymph nodes on final pathology; group 2: two sonographically predicted metastatic lymph node/≤ 2 metastatic lymph nodes on final pathology) undergoing ALND. A negative test outcome was defined as LAD on final pathology. For assessment of sonographic prediction of N0 disease, the FNR was defined as negative preoperative axillary staging but positive SNB, and FPR as positive preoperative axillary staging but negative findings on ALND. For prediction of LAD, FNR was defined as prediction of LAD on preoperative sonography but EAD on ALND, and FPR as prediction of EAD on preoperative sonography but LAD on ALND. For assessment of age-dependent variations of accuracy of axillary sonographic staging, the performed analyses were additionally conducted for the subgroup of patients ≥ 70 years. A multivariate model was used to identify factors associated with high accuracy of axillary sonographic staging in terms of correct identification of patients with LAD. We used the chi-squared test for univariate analysis and stepwise binary logistic regression for multivariate analysis (MVA). Variables that were significant on univariate analysis were included as covariates in the MVA. *P* values < 0.05 were considered significant. G.W. and A.K. performed the statistical analysis using SPSS (IBM SPSS Statistics for Windows, version 27.0. Armonk, NY).

## Results

### Patient and Tumor Characteristics

**Fig. 1:**
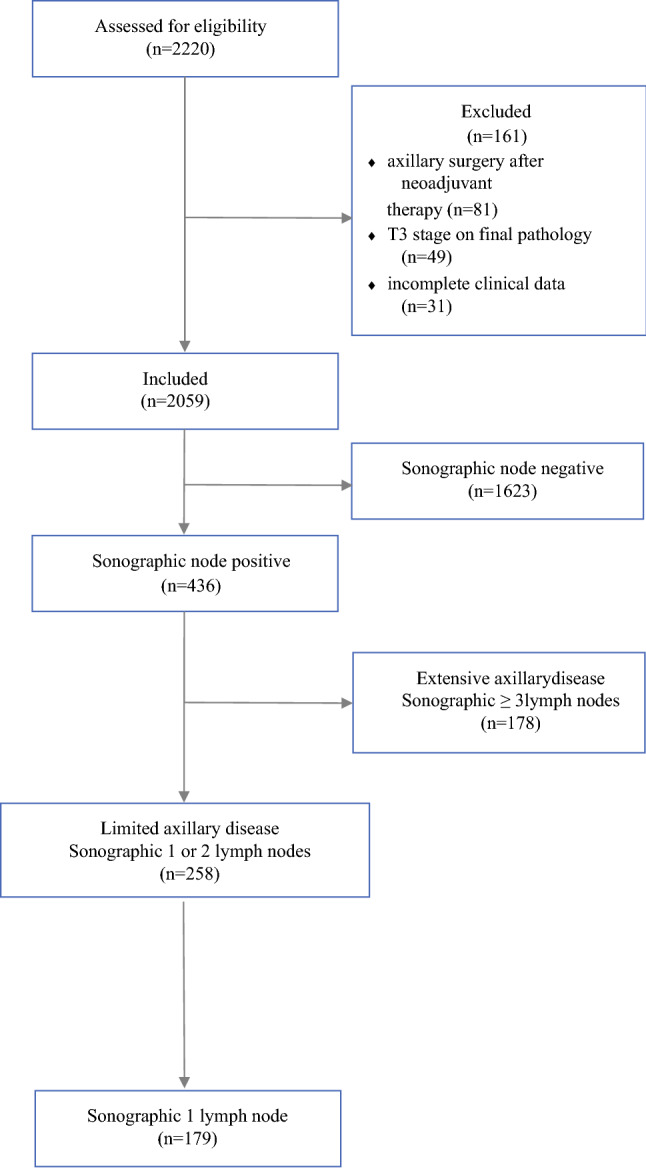
Flowchart of study design

Of 2220 patients, 161 were excluded due to performance of axillary surgery after completion of neoadjuvant chemotherapy (*n* = 81), final pathological staging of T3/T4 or metastatic disease (*n* = 49), and incompleteness of clinical data (*n* = 31), leaving 2059 patients for final analysis (Fig. 1). Patient and tumor characteristics are presented in Table 1. Median age at time of diagnosis was 65 (range 25–92) years, and median body mass index was 25 (range 17–41.1) kg/m^2^. The number of patients 70 years or older was 195, while 10 (0.5%) were *BRCA1*/*2* positive, 31 (1.5%) were negative, and 2018 (98%) were not tested. The results showed that 1165 (57%) patients were diagnosed with T1 and 894 (43%) with T2 breast cancer.

**Table 1:** Patient and tumor characteristics (n = 2059)

	Primary breast cancer
	(*n* = 2059)
	Median (range)
Age (years)	65 (26–95)
Body mass index (kg/m^2^)	25 (17–41.1)

	*n* (%)
Race/ethnicity	
White	2025 (98%)
Afro-American	20 (1%)
Asian	14 (1%)
Histopathologic N stage	
N0	1513 (74%)
N1	393 (19%)
N2	107 (5%)
N3	46 (2%)
Preoperative axillary sonographic status	
Negative	1623 (79%)
Positive	436 (21%)
Histology	
No special type (NST)	1550 (75%)
Lobular	392 (19%)
Other	117 (6%)
Grade	
G1	215 (10%)
G2	1387 (68%)
G3	457 (22%)
Subtype	
Luminal A	919 (45%)
Luminal B	735 (35%)
HER2 positive	189 (9%)
Triple negative	216 (11 %)
Surgery	
Breast-conserving therapy	1433 (70%)
Mastectomy	626 (30%)
Axillary procedure	
Sentinel node biopsy	1513 (74%)
Axillary dissection	436 (21%)
Both	110 (5%)

	Median (range)
Median number of lymph nodes removed	
Sentinel node biopsy	2 (1–11)
Axillary dissection	10 (1–40)
Total	1 (1–40)
Median number of metastatic lymph nodes removed	
Sentinel node biopsy	0 (0–7)
Axillary dissection	0 (0–35)
Total	0 (0–35)

On preoperative axillary sonography, 1623 (79%) patients showed no suspicious axillary nodes and 436 (21%) patients showed suspicious axillary nodes on ultrasound, which were proven to be metastatic by core needle biopsy. Of these 436 patients, 178 (41%) appeared to have EAD (three or more lymph nodes) and 258 (59%) appeared to have LAD (one or two lymph nodes) on preoperative ultrasound. On final pathology, 1513 (74%) patients who underwent SNB had no axillary metastatic lymph node (N0) and 546 (26%) patients who underwent ALND had positive axillary nodes (N1–3). Most patients [*n* = 1550 (75%)] had invasive carcinoma of no special type (NST) histology. Based on immunohistochemical analysis, 919 (45%) patients had luminal A, 735 (35%) patients had luminal B, 189 (9%) patients had HER2/neu positive, and 216 (11%) patients had triple-negative breast cancer. Regarding type of breast and axillary surgery, 1433 (70%) patients had breast-conserving therapy and 626 (30%) mastectomy, 1513 (74%) underwent SNB, 436 (21%) ALND, and 110 (5%) ALND after positive SNB. The median number of lymph nodes removed was 2 (range 1–11) [SNB] and 10 (range 1–40) [ALND], and the median number of metastatic lymph nodes removed was 0 (range 0–7) [SNB] and 0 (0–35) [ALND].

### Accuracy of Sonographic Axillary Staging for Prediction of N0 Disease in Patients Undergoing SNB

Regarding the whole cohort, the accuracy, sensitivity, and specificity for sonographic prediction of absence of nodal involvement were 94% (95% CI 93–95%), 79% (95% CI 75–82%), and 100% (95% CI 99–100%), respectively, with a PPV of 100% (95% CI 99–100%), an NPV of 93% (95% CI 92–94%), an FNR of 21% (95% CI 17–25%), and an FPR of 0% (95% CI 0–0.2%). Among patients with no suspicion of nodal involvement on preoperative ultrasound and one or more positive lymph nodes on final pathology, 17 patients (16%) had micrometastasis (pN1mi) and the other 92 (84%) patients had macrometastasis (N1 disease). For patients aged ≥ 70 years, the accuracy, sensitivity, and specificity for N0 prediction were 95% (95% CI 93–96%), 83% (95% CI 77–88%), and 100% (95% CI 99–100%), respectively, with a PPV of 100% (95% CI 98–100%), an NPV of 93% (95% CI 91–95%), an FNR of 17% (95% CI 12–22%), and an FPR of 0% (95% CI 0–0.7%) (Table 2). There was no subgroup (subtype/T size or combination) in which sonographic prediction of absence of nodal disease showed a clinically insignificant (≤ 10) FNR.

**Table 2:** Accuracy of sonographic axillary staging for prediction of N0 disease in patients undergoing SNB: whole cohort and patients ≥ 70 years

	Whole cohort	Age ≥ 70 years
	Percentages (95% CI)	
Accuracy	94% (93–95%)	95% (93–96%)
Sensitivity	79% (75–82%)	83% (77–88%)
Specificity	100% (99–100%)	100% (99–100%)
Positive predictive value	100% (99–100%)	100% (98–100%)
Negative predictive value	93% (92–94%)	93% (91–95%)
False-negative rate	21% (17–25%)	17% (12–22%)
False-positive rate	0% (0–0.2%)	0% (0–0.7%)

### Accuracy of Sonographic Axillary Staging for Prediction of Limited Axillary Disease in Patients Undergoing ALND

Of 325 patients with histologically confirmed LAD by ALND, 222 (68%) were diagnosed with LAD, 7 (2%) with EAD, and 96 (30%) with N0 disease by preoperative sonographic axillary staging. In 258 (13%) patients, LAD was predicted on preoperative sonographic staging.

For the prediction of LAD in patients with one suspicious lymph node on preoperative sonography (group 1), the accuracy was 94% (95% CI 91–96%), sensitivity was 92% (95% CI 87–95%), and specificity was 96% (95% CI 92–98%), with a PPV of 96% (95% CI 92–98%), an NPV of 92% (95% CI 87–95%), an FNR of 8% (95% CI 5–13%), and an FPR of 5% (95% CI 2–8%). For patients with two suspicious lymph nodes on preoperative ultrasound (group 2), the accuracy, sensitivity, specificity, and NPV decreased to 89% (95% CI 85–93%), 89% (95% CI 84–93%), 89% (95% CI 79–96%), and 73% (95% CI 62–83%), the PPV was 96% (95% CI 92–98%), and the FNR and FPR increased to 11% (95% CI 7–16%) and 11% (95% CI 4–21%) respectively. Among patients with one positive lymph node on preoperative ultrasound, 15 patients had final pathology (three or more metastatic lymph nodes) that would have changed locoregional and systemic treatment.

For patients aged ≥ 70 years with one suspicious lymph nodes on preoperative axillary sonography (group 1), the accuracy, sensitivity, and specificity of LAD prediction were 93% (95% CI 88–97%), 94% (95% CI 86–98%), and 93% (95% CI 85–98%), respectively, and the PPV was 95% (95% CI 88–98%), the NPV was 92% (95% CI 83–97%), the FNR was 7% (95% CI 2–14%), and the FPR was 7% (95% CI 2–16%). For patients aged ≥ 70 years with two suspicious lymph nodes on preoperative ultrasound (group 2), the accuracy [88% (95% CI 81–93%)], sensitivity [90% (95% CI 82–95%)], specificity [80% (95% CI 59–93%)], and NPV [67% (95% CI 57–83%)] decreased while the FNR [10% (95% CI 5–18%)] and FPR increased [20% (95% CI 7–41%)] (Table 3).

**Table 3:** Accuracy of sonographic axillary staging for prediction of LAD (defined as presence of one or two metastatic lymph nodes on final pathology) according to number of suspicious lymph nodes on preoperative ultrasound in patients undergoing ALND (group 1: 1 suspicious lymph node on preoperative sonography/histology ≤ 2 two metastatic lymph nodes; group 2: 2 suspicious lymph nodes on preoperative sonography/histology ≤ 2 metastatic lymph nodes): whole cohort and patients ≥ 70 years

	Group 1 Sonography (1 lymph node)/ histology (≤ 2 lymph nodes)	Group 2 Sonography (2 lymph nodes)/ histology (≤ 2 lymph nodes)
	Percentages (95% CI)	
Whole cohort		
Accuracy	94% (91–96%)	89% (85–93%)
Sensitivity	92% (87–95%)	89% (84–93%)
Specificity	96% (92–98%)	89% (79–96%)
Positive predictive value	96% (92–98%)	96% (92–98%)
Negative predictive value	92% (87–95%)	73% (62–83%)
False-negative rate	8% (5–13%)	11% (7–16%)
False-positive rate	5% (2–8%)	11% (4–21%)
Age ≥ 70 years		
Accuracy	93% (88–97%)	88% (81–93%)
Sensitivity	94% (86–98%)	90% (82–95%)
Specificity	93% (85–98%)	80% (59–93%)
Positive predictive value	95% (88–98%)	95% (88–98%)
Negative predictive value	92% (83–97%)	67% (57–83%)
False-negative rate	7% (2–14%)	10% (5–18%)
False-positive rate	7% (2–16%)	20% (7–41%)

### Multivariate Analysis for Factors Associated with High Accuracy of Sonographic Identification of Limited Axillary Disease

On univariate analysis, correct identification of patients with histologically confirmed LAD by ALND was associated with the HER2/neu subtype [OR 0.32 (95% CI 0.11–0.94), *p* = 0.04], T2 stage [OR 0.49 (95% CI 0.34–0.73), *p* ≤ 0.01], G3 [OR 0.23 (95% CI 0.07–0.74), *p* ≤ 0.01], lymphovascular invasion [OR 0.36 (95% CI 0.24–0.54), *p* ≤ 0.01], and the number of suspicious lymph nodes on preoperative sonography [OR 0.08 (95% CI 0.05–0.13), *p* ≤ 0.01]. On multivariate analysis, including these parameters, the number of suspicious lymph nodes on preoperative ultrasound was the only parameter that correlated inversely with correct prediction of LAD [OR 0.01 (95% CI 0.01–0.93), *p* ≤ 0.01]. Similar results were obtained for the subgroup of patients ≥ 70 years (Table 4).

**Table 4:** Uni- and multivariate analyses of factors associated with correct preoperative sonographic prediction of limited histopathologic axillary disease (one or two metastatic lymph nodes): for the whole cohort and patients ≥ 70 years; NST, no special type

	Univariate analysis		Multivariate analysis	
	Odds ratio (95% CI)	*p*	Odds ratio (95% CI)	*p*
Whole cohort				
Body mass index	1.00 (0.99–1.01)	0.69		
Age	0.99 (0.97–1.00)	0.06		
Histology		0.69		
NST versus invasive lobular	0.26 (0.01–25.96)	0.59		
NST versus others	0.19 (0.01–19.29)	0.48		
Subtype		0.11		
Luminal A versus luminal B	0.72 (0.48–1.08)	0.11		
Luminal A versus HER2/neu	0.32 (0.11–0.94)	0.04	2.36 (0.26–21.12)	0.44
Luminal A versus triple negative	0.68 (0.33–1.39)	0.29		
T stage				
T1 versus T2	0.49 (0.34–0.73)	≤ 0.01	1.61 (0.56–4.67)	0.61
Grade		0.01		0.98
G1 versus G2	0.39 (0.13–1.26)	0.12	1.37 (0.03–62.28)	0.87
G1 versus G3	0.23 (0.07–0.74)	≤ 0.01	1.87 (0.54–6.43)	0.32
Number of affected lymph nodes	0.08 (0.05–0.13)	≤ 0.01	0.01 (0.01–0.93)	≤ 0.01
Lymphovascular invasion (yes/no)	0.36 (0.24–0.54)	≤ 0.01	0.53 (0.19–1.48)	0.23
Age ≥ 70 years				
Body mass index	1.01 (0.99–1.04)	0.37		
Age	1.01 (0.95–1.06)	0.80		
Histology		0.08		
NST versus invasive lobular	0.29 (0.01–25.96)	0.59		
NST versus others	0.19 (00.01–19.29)	0.48		
Subtype		0.11		
Luminal A versus luminal B	0.72 (0.39–1.33)	0.29		
Luminal A versus HER2/neu	0.20 (0.02–1.79)	0.15		
Luminal A versus triple negative	0.89 (0.31–2.56)	0.83		
T stage				
T1 versus T2	0.52 (0.29–0.94)	0.03	1.61 (0.56–4.67)	0.38
Grade		0.01		0.61
G1 versus G2	0.60 (0.09–3.72)	0.59	1.37 (0.03–62.28)	0.87
G1 versus G3	0.37 (0.06–2.43)	0.30	1.87 (0.54–6.44)	0.32
Number of affected lymph nodes	0.07 (0.03–0.15)	≤ 0.01	0.01 (0.01–0.03)	≤ 0.01
Lymphovascular invasion (yes/no)	0.39 (0.22–0.71)	≤ 0.01	0.53 (0.19–1.48)	0.23

## Discussion

In this retrospective analysis of a prospectively maintained service database, we found high rates for preoperative identification of limited axillary disease (LAD) defined as one or two metastatic nodes on final pathology, in patients with early-stage breast cancer in whom one metastatic lymph node was predicted on preoperative ultrasound and who underwent ANLD. With an FNR of 8% for this prediction, these findings are comparable to those of SNB (5–9%).^11^ However, the FNR in the present study might have greater clinical implications since it means that these patients had EAD, whereas the FNR in SNB patients only means these patients have additional nodal disease. The actual percentage of false-negative SNB patients with EAD is not known, but in the original Z0011 population only 27.3% patients who underwent ALND after a positive SNB had additional metastasis in lymph nodes removed by ALND, and 21% of these had EAD.^5^

Two smaller studies have demonstrated the feasibility of preoperative LAD prediction.^7,16^ Although the authors did not provide statistical measures on test validities, they reported a high probability of having two or fewer metastatic lymph nodes on final pathology in patients with one abnormal node on preoperative axillary sonography and concluded that these patients should be offered SNB.^16^ Large clinical trials have shown that systemic therapy decisions for postmenopausal women with early-stage, hormone receptor-positive breast cancer and LAD should be based on genomic assays, rather than on the number of affected lymph nodes.^17–19^ In these patients, the benefit of identifying additional metastatic lymph nodes by SNB after preoperative image-based LAD confirmation seems questionable. Given this lack of additional therapeutic implications and the low FNR rate for patients with one preoperatively suspicious lymph node in this study, SNB omission might be an option worth considering in these patients. However, our results indicate that this approach cannot be recommended for patients with up to two suspicious lymph nodes on preoperative sonography. Although 89% of patients with histopathologically confirmed LAD were correctly identified via ultrasound, 11% of patients with one or two suspicious nodes on preoperative sonography were diagnosed false negative, and axillary surgery omission would have constituted undertreatment. Similarly, accuracy of LAD prediction in patients fulfilling the Z0011 criteria seemed to be dependent on the number of suspicious nodes identified by preoperative sonography in several smaller studies.^7,8,16^ In our analysis, the number of preoperatively suspicious lymph nodes was the only factor associated with correct prediction of LAD. Our findings add to accumulating evidence demonstrating an association between an increasing number of suspicious lymph nodes on preoperative ultrasound and a higher number of metastatic nodes on final histology.^20–24^ Thus, and following widespread implementation of the Z0011 approach into clinical practice, many imaging studies have focused on preoperative extensive axillary disease and reported PPVs of up to 92.9%, supporting the high accuracy of prediction of heavy nodal burden, and imply that these patients can safely proceed to axillary dissection without SNB.^25,26^

A collective in whom the benefit of SNB has been the focus of current clinical research are patients with clinical node-negative disease on preoperative imaging. As these patients represent more than 70% of primary breast cancer patients, omission of SNB in these patients would be a significant contribution to deescalation of axillary surgery.

In this study, 20% of preoperative sonographic N0 diagnoses were false negative. Similar results were reported in a metaanalysis assessing preoperative sonographic staging of the axilla including ultrasound-guided biopsy of suspicious nodes in 9212 patients with primary breast cancer. The authors found a sensitivity of 50% and an FNR of 25% regarding prediction of nodal involvement, concluding that preoperative axillary staging could not replace SNB in these patients.^27^

The use of SNB in older patients has been questioned lately, and current recommendations advise that SNB in patients ≥ 70 years with early-stage, hormone receptor-positive, HER2-negative breast cancer and no palpable axillary lymph node treated with endocrine therapy can be considered individually with regard to its impact on radiation recommendations and systemic therapy decisions.^28–30^ A National Cancer Database study compared patients aged ≥ 70 years, with clinical negative axilla, where 99,764 patients underwent axillary surgery and 31,531 did not. Beside an improved overall survival in the group of patients who were treated with axillary surgery, the authors found 14% of the clinical node-negative patients to be node positive on final pathology, which is well in line with our false-negative rate of 16%.^31^ As these sonographically N0 patients would not receive postoperative axillary radiation, SNB omission might lead to undertreatment in these cases. Several ongoing studies are exploring this issue and will improve the selection of elderly patients in whom omission of axillary surgery can be safely performed and help to identify clinical features that refine a low-risk collective in this setting.^32,33^

The generalizability of our findings is limited, given the variability in ultrasound image acquisition and interpretation, although the inclusion of data from two centers increases their reliability. The transfer of the findings to other populations has to be done cautiously as the collective assessed mostly comprised screening patients, with low rates of axillary nodal burden and moderate numbers of involved lymph nodes. However, the therapeutic consequences identified in the Z0011 trial are transferable to our sample, which was similar to the Z0011 population (medians of two and one metastatic lymph nodes).^5^

In patients with early-stage breast cancer fulfilling the Z0011 criteria with one abnormal axillary lymph node identified by preoperative sonography who underwent ALND, the prediction of limited axillary disease on final pathology was highly accurate, with a false-negative rate comparable to that of sentinel node biopsy. These findings imply that omission of sentinel node biopsy might be an option that can be considered in these patients. With the ability to correctly distinguish limited from extensive nodal disease, axillary imaging represents a key clinical decision-making tool for management of the axilla and can be used to further individualize and deescalate surgical staging.
